# Bergmeister’s papilla in a young patient with type 1 sialidosis: case report

**DOI:** 10.1186/s12886-020-01628-1

**Published:** 2020-08-31

**Authors:** Settimio Rossi, Carlo Gesualdo, Antonio Tartaglione, Leonilda Bilo, Antonietta Coppola, Francesca Simonelli

**Affiliations:** 1 Eye Clinic, Multidisciplinary Department of Medical, Surgical and Dental Sciences, University of Campania L. Vanvitelli, 80131 Naples, Italy; 2 Department of Neuroscience and Reproductive and Odontostomatological Sciences, Federico II University, Naples, Italy

**Keywords:** Sialidosis, Bergmeister’s papilla, Optical coherence tomography

## Abstract

**Background:**

Sialidosis is a rare genetic lysosomal storage disorder caused by a deficit of neuraminidase enzyme activity. Patients with sialidosis present various neurological disorders such as: myoclonic epilepsy and hypotonia, often associated with visual impairment. A typical aspect of sialidosis is the finding of a macular cherry-red spot on ocular fundus examination. In this paper we describe a unilateral case of Bergmeister’s papilla (BP) in a young female patient suffering from type 1 sialidosis.

**Case presentation:**

A 28-year-old young woman suffering from type 1 sialidosis, confirmed by previously described compound heterozigosity Leu91Arg and Gly328Ser on N-acetyl-alpha-neuraminidase − 1 (NEU1) gene, underwent an opthalmological examination at the Eye Clinic of the University of Campania L. Vanvitelli, for bilateral visual deterioration. The patient was suffering from myoclonic epilepsy with hypotonia and severe motor disability. Fundoscopic examination showed a typical macular cherry-red spot with retinal pigment epithelium dystrophy in the middle periphery, in both eyes. Furthermore, in the left eye (OS), a vitreous thickening was observed in the nasal sector of the optic disc, remnant of fetal vasculature on the optic disc (Bergmeister’s papilla). Optical coherence tomography (OCT) showed, in both eyes, a thickening of the ganglion cell layer (GCL) with a hyperreflective opacity as a cap on the left optic disc.

**Conclusions:**

In our paper we have described, for the first time in literature, a case of BP in a patient with type 1 sialidosis. The detection of BP with thickening of the peripapillary vitreous by SD-OCT is useful in monitoring any vitreo-retinal change that could cause future visual deterioration.

## Background

Sialidosis is a rare genetic lysosomal storage disease caused by mutations in the N-acetyl-alpha-neuraminidase − 1 (NEU1) gene (chromosome 6p21), encoding the lysosomal enzyme neuraminidase, which triggers the degradation of sialyloligosaccharides in lysosomes. The mutations cause a decrease in enzymatic activity and, consequently, an accumulation of sialyloligosaccharides in the tissues [1]. Two forms of the disease have been identified: type 1 sialidosis (ST-1) with later onset and better prognosis, and type 2 sialidosis (ST-2), a more severe early-onset condition. The main characteristics of type 1 sialidosis are: myoclonic epilepsy, cerebellar ataxia, hypotonia and progressive visual decline. Instead, ST-2 is characterized by early onset, sometimes it can be congenital, and causes facial dysmorphism, bone dysplasia or psychomotor retardation. Almost all patients with sialidosis have a cherry-red spot in the macular region, which is therefore an important distinctive element in the differential diagnosis with other neurological metabolic disorders. Other possible ocular features, prevalent in ST-1, are: nystagmus, night blindness and in some cases the presence of corneal opacities [2–6]. In this paper we describe, for the first time, a case of ST-1 with the typical macular cherry-red spot, associated with a residue of hyaloid artery on the left optic disc (Bergmeister’s papilla).

## Case presentation

**Fig. 1:**
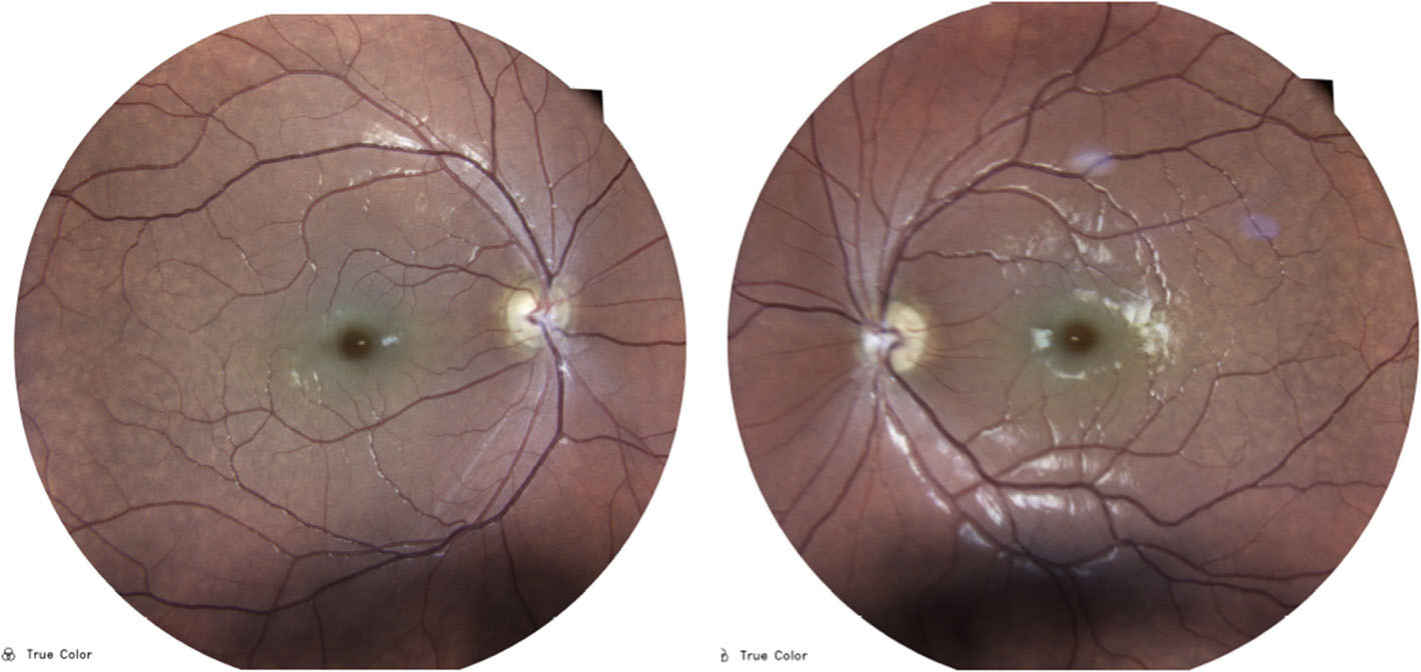
Fundus photographs shows in both eyes the typical macular cherry-red spot with retinal pigment epithelium dystrophy in the middle periphery

A 28-year-old young woman suffering from type 1 sialidosis, underwent a complete ophthalmological assessment in October 2019, at the Eye Clinic of the University of Campania L. Vanvitelli, complaining of a progressive decline of visual acuity in recent months. Polymerase chain reaction (PCR) and Sanger sequencing analysis of NEU1 gene disclosed an already described compound heterozygosity: Leu91Arg and Gly328Ser [7, 8]. The patient suffered from myoclonic epilepsy with hypotonia and severe motor disability, from approximately the age of 13 years. Best corrected visual acuity (BCVA) measured with Early Treatment Diabetic Retinopathy Study (ETDRS) chart at 2 m was: 45 letters (20/63) in the right eye (OD) and 33 letters (20/100) in the left eye (OS). Slit lamp examination showed a slight diffuse opacity of the corneal endothelium in both eyes, with incipient lens cortical opacities. There was no nystagmus. Examination of the ocular fundus and retinography performed with True Color confocal ophthalmoscope EIDON, showed a macular cherry-red spot bilaterally, with diffuse dystrophy of the retinal pigment epithelium in middle periphery. In the right eye the optic disc was normal, while in the left eye, on the nasal portion of the optic disc, there was a whitish area of vitreous thickening, residual from the fetal vascularization on the optic disc (Bergmeister’s papilla) (Fig. 1).

**Fig. 2:**
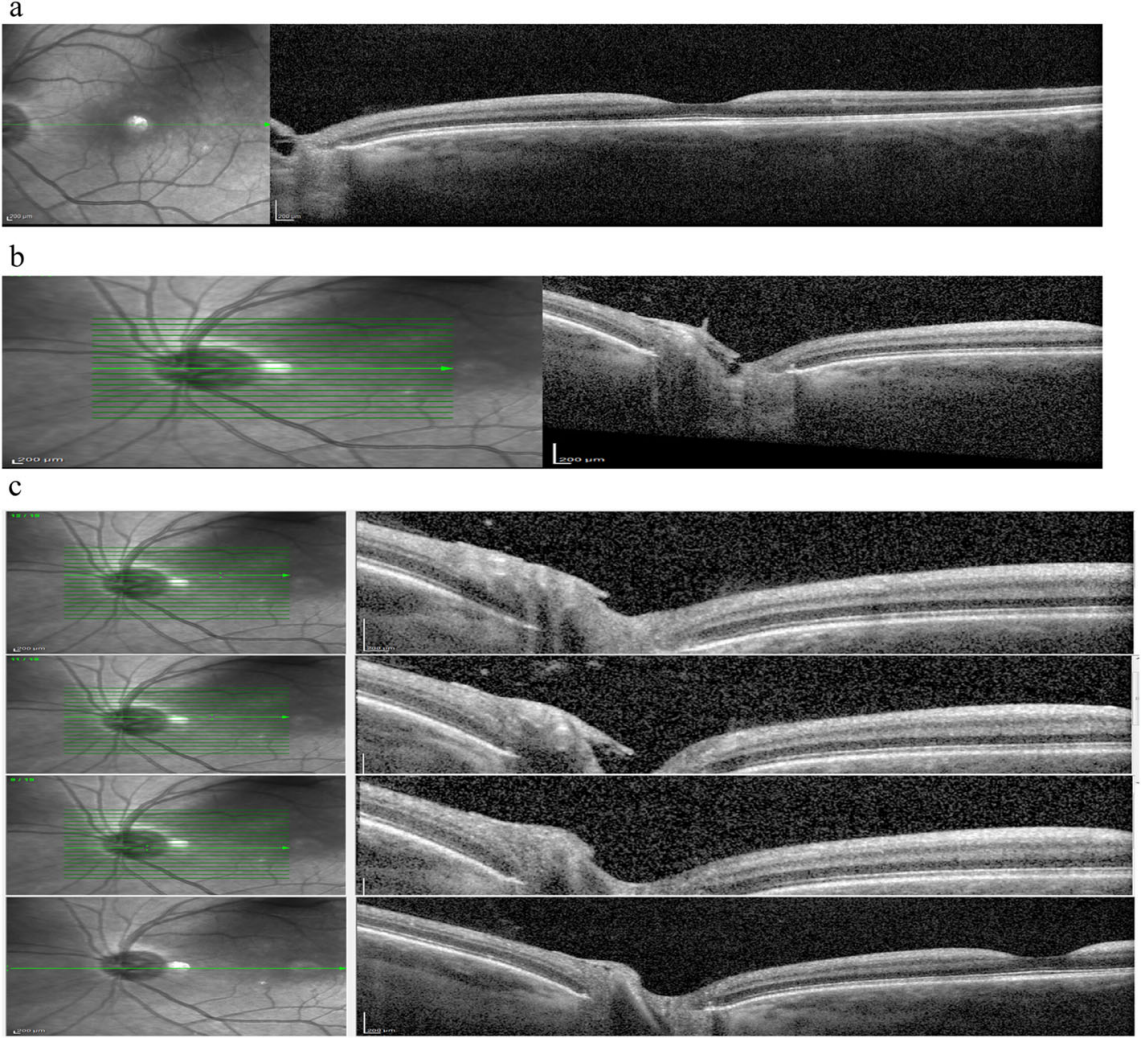
**a** Spectral domain optical coherence tomography shows a hyperreflectivity with thickening of ganglion cell layer. **b** Spectral domain optical coherence tomography of the optic nerve shows the presence of a hyperreflective opacity as a cap on the left optic disc. **c** Serial B-scan Optical coherence tomography (OCT) images focusing the optic disc

An optical coherence tomography (SD-OCT), with Spectralis (Heidelberg Engineering), was also performed, and showed a thickening with increased reflectivity of ganglion cell layer (GCL) in both eyes (Fig. 2a), in accordance with data already described [9, 10]. Furthermore the SD-OCT has shown a hyperreflective opacity as a cap on the left optic disc. (Fig. 2b, c).

## Discussion and conclusions

Sialidosis is a rare autosomal-recessive lysosomal storage disease characterized by the progressive accumulation of sialyloligosaccharides in the tissues, especially in the brain and in the retinal nerve fibers [2, 3]. Both forms of sialidosis identified so far (ST-1, ST-2), are due to mutations of NEU1 gene located on 6p21.33, which result in a deficit in the activity of the lysosomal enzyme neuraminidase, and consequent accumulation of sialyl oligosaccharides [1]. We have described, for the first time, a case of BP in a young patient with ST-1. The Bergmeister’s papilla is is a rare congenital anomaly of the optic disc, characterized by the persistence of residues of the hyaloid artery on the optic disc, in the form of an epipapillary glial membrane, which can occlude the papilla or part of it, making it sometimes difficult to visualize. In healthy fetuses, the hyaloid artery which is responsible for the vascularization of the crystalline lens during embryo development through the Cloquet’s canal, progressively regresses from 10 weeks of gestation, until it disappears at birth. Failure to regress the hyaloid artery can be partial or complete; the residue of the anterior portion on the posterior lens capsule is called Mittendorf’s dot, while the posterior residue on the optic disc is called Bergmeister’s papilla, and is usually made up of glial tissue. Complete persistence of the hyaloid artery extending from the papilla to the lens, which could cause sudden vitreous bleeding, is very rare. BP, in most cases, is an occasional finding, which has no clinical impact. However, in the most severe forms it can be associated with cataracts, persistence of the primitive vitreous, microphthalmia, vitreous hemorrhages and sometimes tractional retinal detachment, due to contraction of the residual fibrovascular tissue [11–13]. Therefore, in case of suspected BP, careful monitoring of eventual vitreous thickening in the peripapillary areas, both by examining the ocular fundus, and especially by SD-OCT, is of considerable importance. In this case report, despite the difficulty of execution, the role of SD-OCT has been fundamental, to identify a hyperreflective opacity as a cap on the left optic disc, remnant of fetal vasculature, with adjacent vitreo-retinal adhesion, that must be monitored. Therefore, our case underlines the importance of performing SD-OCT in neurological pathologies characterized by cherry-red macular spot such as sialidosis, allowing to better visualize a congenital anomaly of the optic disc, not clearly visible with ocular fundus examination alone. Similar cases presenting remnant hyaloid artery should be monitored over time being a possible cause of future visual deterioration.

## Data Availability

The datasets used and/or analysed during the current study are available from the corresponding author on reasonable request.

## Abbreviations

BCVA: Best corrected visual acuity
BP: Bergmeister’s papilla
ETDRS: Early treatment diabetic retinopathy study
GCL: Ganglion cell layer
NEU1: Neuraminidase1
OD: Right eye
OS: Left eye
PCR: Polymerase chain reaction
SD-OCT: Spectral domain – optical coherence tomography
ST-1: Sialidosis-1
